# Biosimilars in the United States: Considerations for Oncology Advanced Practitioners

**DOI:** 10.6004/jadpro.2015.6.2.3

**Published:** 2015-03-01

**Authors:** Kelley D. Mayden, Paul Larson, Danielle Geiger, Holly Watson

**Affiliations:** 1 Southwest Virginia Cancer Center, Norton, Virginia;; 2 Fletcher Allen Health Care, Burlington, Vermont;; 3 Nebraska Cancer Specialists, Omaha, Nebraska;; 4 Amgen Inc., Thousand Oaks, California

## Abstract

Biosimilars will enter the US market soon, potentially lowering costs and increasing patient access to important oncology biologics. Biosimilars are highly similar, but not identical, to their reference product. Subtle variations arise due to their inherent complexity and differences in manufacturing. Biosimilars are not generic drugs. They will be approved through a separate US regulatory pathway—distinct from conventional biologics license applications—based on analytic and clinical studies demonstrating no clinically meaningful differences from the reference product. As policies on US biosimilars evolve, it is important that advanced practitioners receive comprehensive, ongoing education on them, particularly regarding differences from small-molecule drugs; their approval pathways vs. conventional regulatory pathways; evaluation of quality, safety, and efficacy; safety monitoring; and product identification to facilitate accurate safety reporting. Advanced practitioners will play a key role in educating nurses and patients on biosimilars. Nurse education should highlight any differences from the reference product (e.g., approved indications and delivery devices) and should emphasize assessment of substitutions, monitoring for adverse events (e.g., immune reactions), and the need for precise documentation for safety reports. Patient education should address differences between the biosimilar and reference product in administration, handling and storage, and self-monitoring for adverse events.

Biologics are an essential part of cancer treatment and provide opportunities for the delivery of targeted therapy and supportive care (Table 1). United States patents for some first-generation biologics used in cancer care may soon expire, according to one source (Generics and Biosimilars Initiative, 2013), creating opportunities for the development of similar versions called biosimilars. Biosimilars are expected to become available in the United States soon and may lower health-care costs by stimulating price competition for biologics, ultimately increasing patient access to biologics (IMS Health, 2011; Kozlowski, Woodcock, Midthun, & Sherman, 2011; Zelenetz et al., 2011). Biologics with likely biosimilar development in the United States include products for cancer treatment (e.g., bevacizumab, cetuximab, rituximab, and trastuzumab) and for supportive care (e.g., epoetin alfa, filgrastim, and pegfilgrastim). According to US law, a biosimilar is defined as a biologic product that is highly similar to its reference product (i.e., the original biologic that the biosimilar product is intended to copy; Table 1; Biologics Price Competition and Innovation, 2009). A biosimilar may have minor differences in clinically inactive components. However, it should have no clinically meaningful differences from its reference product in terms of safety, purity, and potency (Biologics Price Competition and Innovation, 2009).

**Table 1:**
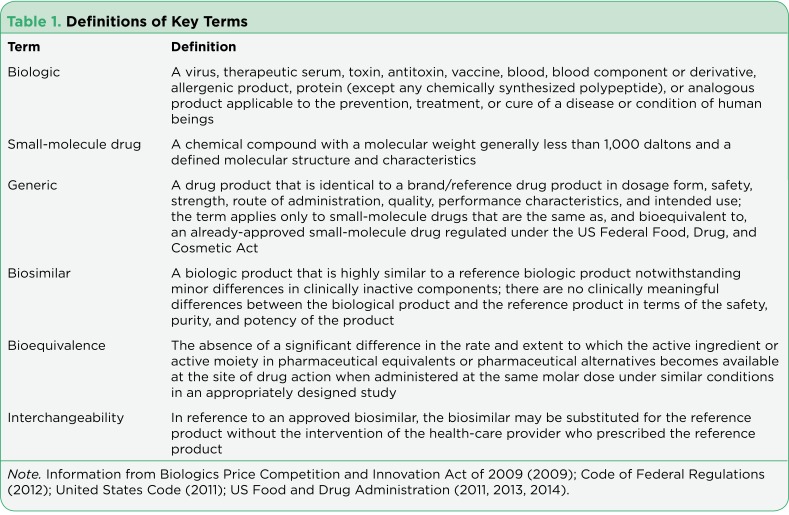
Definitions of Key Terms

This article reviews important considerations for advanced practitioners in preparation for the emergence of oncology biosimilars, including key properties of biosimilars and how they differ from generic drugs; the regulation of biosimilars; the evaluation of biosimilar quality, safety, and efficacy; postapproval safety monitoring; and the naming of biosimilars in relation to identification and tracing of adverse events (AEs) to the correct product. This review also highlights the need for ongoing education of advanced practitioners (APs) with regard to biosimilars and the central role APs will play in their prescription, patient management, and education of oncology nursing professionals and patients about these emerging agents.

## BIOSIMILARS ARE NOT GENERICS

Although identical copies of a small-molecule drug can be manufactured to make a generic version of the drug (Table 1), identical copies of an original biologic cannot be made. Biosimilars are not generics and can be considered a new class of biologics (Zelenetz et al., 2011). The distinction between biologics and small-molecule drugs stems from differences in their fundamental properties (Table 2). Small-molecule drugs are organic molecules with a single, defined structure. In contrast, biologics are many times larger than chemical-based small-molecule drugs, and as protein-based drugs, biologics are composed of hundreds of amino acid subunits, with three-dimensional protein structures that may further twist and bend to form larger, folded structures (Declerck, 2012; Lee, Litten, & Grampp, 2012; Sekhon & Saluja, 2011; Zelenetz et al., 2011).

**Table 2:**
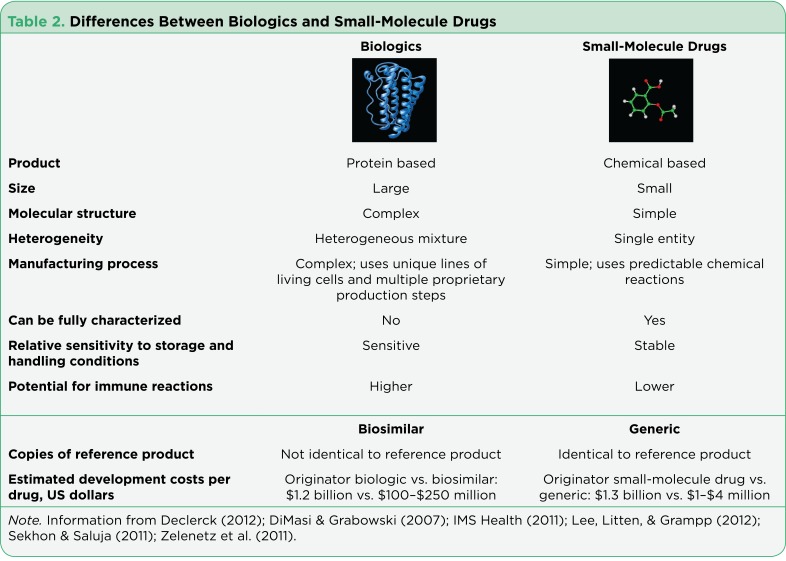
Differences Between Biologics and Small-Molecule Drugs

The proteins of biologics may also undergo modifications (e.g., variations in sugars attached to the protein) that result in a mixture of structures for a particular biologic (Declerck, 2012; Lee et al., 2012; Sekhon & Saluja, 2011; Zelenetz et al., 2011). Because biologics are large, complex proteins, they are more sensitive than small-molecule drugs to changes in storage and handling conditions that can result in denaturation and degradation and an increase in the potential for immune reactions in patients (Declerck, 2012; Lee et al., 2012; Sekhon & Saluja, 2011; Zelenetz et al., 2011).

Biologics and small-molecule drugs also differ in how they are manufactured. Small-molecule drugs are synthesized by predictable chemical reactions. These reactions can be reproduced to make identical copies (i.e., generics) of small-molecule drugs, which can be fully characterized by analytical methods (Table 2; Declerck, 2012; Sekhon & Saluja, 2011; Zelenetz et al., 2011). In contrast, biologics are made using living cells and production processes (Figure) that result in heterogeneous mixtures of proteins that cannot be fully characterized by current analytic techniques (Casadevall et al., 2013; Declerck, 2012; Lee et al., 2012; Sekhon & Saluja, 2011; US Food and Drug Administration [FDA], 2012a; Zelenetz et al., 2011). Differences among manufacturers in these processes result in biosimilars that may have subtle structural variations from the reference product (Casadevall et al., 2013). The clinical effects, if any, resulting from differences between a biosimilar and its reference product may not be known when a biosimilar is approved or after manufacturing processes change, requiring ongoing safety monitoring.

**Figure 1:**
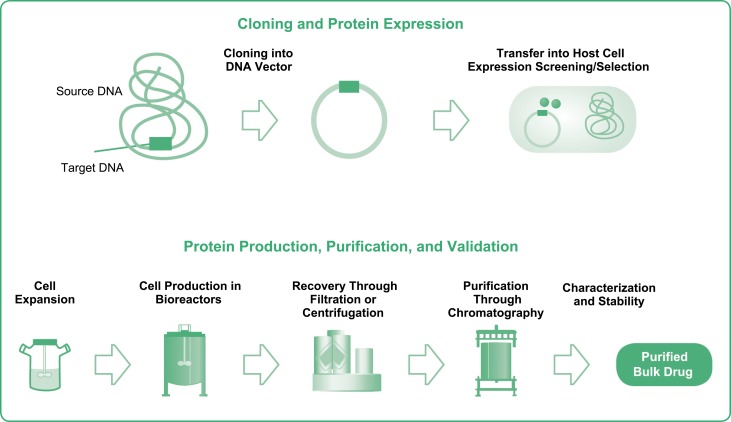
Biologics manufacturing processes. Manufacturing biologics includes a number of steps that may vary among manufacturers. This variance may lead to differences between a biosimilar and its reference product that cannot be fully characterized with currently available analytic techniques. Smallmolecule drugs, by contrast, are synthesized using reproducible chemical reactions to make identical copies (generics) that can be fully characterized with available analytic methods. Used with permission from Mellstedt, Niederwieser, & Ludwig (2008).

## REGULATION OF BIOSIMILARS

A generic drug is approved through an Abbreviated New Drug Application pathway established by the FDA that requires demonstration that the proposed generic is identical to its reference drug in active ingredient, strength, dosage form, route of administration, and conditions of use and that it is bioequivalent to the reference drug in healthy volunteers (FDA, 2011). This pathway is considered abbreviated because manufacturers of generic drugs are not typically required to conduct preclinical and clinical trials to establish safety and efficacy (Sekhon & Saluja, 2011).

In recognition of the differences between biosimilars and generic small-molecule drugs, the European Medicines Agency (EMA), the World Health Organization (WHO), and the FDA have developed distinct approval pathways for biosimilars (European Medicines Agency, 2005; US FDA, 2012a, 2012b; WHO, 2009). The EMA was the first regulatory agency to develop biosimilar guidelines and an approval pathway that has been generally considered to be successful (Directive 2004/27/EC, 2004; EMA, 2005, 2006a, 2006b). The regulatory approval process in the European Union includes specific requirements to demonstrate the comparability of the candidate biosimilar to the reference biologic product in terms of quality and clinical pharmacokinetic and pharmacodynamic evaluations as well as clinical safety and efficacy assessed through comparative clinical trials. Twelve distinct biosimilars have been approved in the European Union through this pathway since 2006 (European Public Assessment Reports: Biosimilars, 2015). Currently, 11 biosimilars are authorized for marketing in the European Union: 2 distinct erythropoietin products (5 brands), 4 distinct granulocyte colony-stimulating factor products (8 brands), 1 growth hormone product (1 brand), 1 monoclonal antibody against tumor necrosis factor–alpha (2 brands), 2 follicle-stimulating hormone products (2 brands), and 1 insulin glargine (1 brand; European Public Assessment Reports: Biosimilars, 2015). A growth hormone product and a granulocyte colony-stimulating factor product were withdrawn following approval for commercial reasons (European Public Assessment Reports: Biosimilars, 2015). As of 2013, no specific safety issues had been identified for approved and marketed biosimilars in the European Union, suggesting that the rigorous clinical testing requirements and review and approval processes in place have been effective thus far (European Medicines Agency, 2013).

Other regulatory agencies around the world, including the FDA, have drawn on the European Union experience with biosimilars to develop their own regulatory pathways. In the United States, the legal basis for a biosimilar approval pathway was established through an amendment of the Public Health Service Act (PHSA) by the Biologics Price Competition and Innovation (BPCI) Act of 2009 (Biologics Price Competition and Innovation, 2009). The BPCI Act is part of the Patient Protection Affordable Care Act signed into law in 2010 and upheld in a US Supreme Court decision in June 2012. The BPCI Act created the biosimilar biologics license application pathway, allowing biosimilar applications to be submitted under section 351(k) of the PHSA (Biologics Price Competition and Innovation, 2009).

The framework for the approval of a biosimilar in the United States is described in draft FDA guidance documents released for public review in 2012 and reviewed here (FDA, 2012a, 2012b, 2012c). Some biologics manufacturers may choose to seek approval of a product similar to a previously approved biologic through a conventional 351(a) biologics license application (e.g., tbo-filgrastim) rather than through the 351(k) biosimilar pathway (FDA, 2012d). Such products would not be considered biosimilars from a US regulatory perspective, although they may have been approved as biosimilars in other regulator regions (e.g., European Union), potentially leading to confusion among some health-care professionals regarding applicable requirements (e.g., interchangeability, documentation, and prescriber notification of pharmacy-level substitutions).

## EVALUATION OF QUALITY, SAFETY, AND EFFICACY OF BIOSIMILARS

The FDA will evaluate biosimilarity based on differences between the proposed biosimilar and its reference product using several parameters as assessed by analytic assays, preclinical studies, and clinical trials (FDA, 2012a, 2012b, 2012c). For the evaluation of quality, the FDA recommends analytic studies that compare the molecular structure, protein modifications (e.g., difference in attached carbohydrates), activity, and purity of the proposed biosimilar vs. its reference product (FDA, 2012b). These assessments are critical not only for characterizing the proposed biosimilar but also for identifying the potential structural or functional differences that may result from using different cell lines or manufacturing processes than those of the reference product (FDA, 2012b).

The FDA recommends preclinical studies that compare the toxicity of the proposed biosimilar and reference product in animal models, followed by clinical studies that compare pharmacokinetics, pharmacodynamics, and immunogenicity (FDA, 2012a). Additional head-to-head clinical trials to compare clinical safety and effectiveness between the proposed biosimilar and the reference product are recommended if biosimilarity is still uncertain (FDA, 2012a). When approved and administered according to their labeling, biosimilars will be considered to have safety and efficacy profiles that are highly similar to the reference biologic product.

Current FDA guidelines will allow some biosimilars to be further designated as interchangeable with the reference product, allowing substitution by the pharmacist without the prescriber’s intervention or knowledge (FDA, 2012b, 2012c). To be designated as interchangeable, a biosimilar is "expected to produce the same clinical result as the reference product in any given patient" and if "administered more than once to an individual, the risk in terms of safety or diminished efficacy of alternating or switching between use of the [biosimilar] and the reference product is not greater than the risk of using the reference product without such alternation or switch" (Biologics Price Competition and Innovation, 2009). The FDA has not yet defined the clinical study requirements for demonstrating interchangeability with the reference product, requiring ongoing awareness and education of health-care providers as policies on interchangeability continue to develop. Biologics that are similar to a previously approved biologic could alternatively be approved through a conventional biologics license application; however, such products will not be eligible for interchangeability status because they will not have been approved via the biosimilar pathway; this scenario underscores the need for ongoing education as new biologics become available in the United States via either the conventional biologic license application or biosimilar pathway.

Ongoing safety monitoring after approval (pharmacovigilance) to accurately attribute AEs to the administered product is important for all biologics, including biosimilars (FDA, 2012a). Accurate pharmacovigilance requires that the specific administered products be easily identified in AE reports (Casadevall et al., 2013; Kozlowski et al., 2011). Difficulties in tracing AEs could arise if a biosimilar shares the same nonproprietary name as its reference product or other biosimilars. Current policies for biosimilar naming differ between regions worldwide (Australian Government Department of Health Therapeutic Goods Administration, 2013; Bogaert, Lietzan, & Sim, 2011; WHO, 2013a). The WHO has noted that assigning biosimilars identical nonproprietary names may lead to unintentional switching (WHO, 2013a, 2013b). The WHO is evaluating different approaches to address the naming of biologics globally, including assigning biosimilars a name with a unique code added to the root nonproprietary name of the reference product (e.g., nonproprietary name-xyz; WHO, 2013a).

Although the FDA has not yet defined its approach to naming biosimilars, it should be determined and communicated to health-care professionals who administer biologics before the first biosimilar is approved to ensure proper documentation of administered products and accurate tracing of any related AEs to the correct product. A unique nonproprietary name for each biologic, whether approved through a biologics license application or the biosimilar pathway, facilitates effective tracing of AEs to the correct product by allowing the specific administered product(s) to be clearly identified in AE reports (Casadevall et al., 2013). It will be important for advanced practitioners to understand the need to accurately and precisely identify products to ensure patient safety and efficient pharmacovigilance.

## INCORPORATION OF BIOSIMILARS INTO ADVANCED ONCOLOGY PRACTICE IN THE UNITED STATES: THE IMPORTANCE OF EDUCATION

The approval of biosimilars may offer potential benefits to patients in terms of increased access to important biologic medications and reduced costs due to price competition. However, it will be critical for prescribers, nurses, and patients to be educated about biosimilars as this new class of biologics is introduced into oncology practice in the United States.

A survey of 277 health-care providers (including physicians, nurses, and pharmacists) conducted by the National Comprehensive Cancer Network (NCCN) showed that there was a suboptimal level of understanding of biosimilars and their regulation (Zelenetz et al., 2011). Among the respondents, nearly half of the 71 nurses (44%) indicated that they were not at all familiar with biosimilar developments, including legislation creating the US biosimilar approval pathway. In addition, approximately one-third (31%) indicated that they would need more information before deciding on their interest level for prescribing, dispensing, or administering biosimilars in their oncology practice setting (Zelenetz et al., 2011).

More recently, a similar survey was conducted among 470 prescribing physicians in 5 European countries (Dolinar & Reilly, 2014). Although the survey was conducted among a different population of respondents, the results turned out to be strikingly similar. More than half of the respondents (54%) reported being familiar with biosimilars—but with only a basic understanding of them. Another 20% of respondents were unable to define biosimilars, and 4% had not previously heard of biosimilars. Additional studies to better assess the extent of practitioners’ knowledge and further identify knowledge gaps and educational needs may be of benefit.

Advanced practitioners can play a key role in educating nurses on these important issues and in providing access to clinical data on biosimilars to support their incorporation and appropriate use in oncology practice. Consequently, knowledge of biosimilar-related principles and policies should be included in the needs assessments and incorporated into educational planning for all oncology nurse professionals.

Although nursing professionals are familiar with administering generic drugs, continuing education will be critical for successful incorporation of biosimilars into oncology practice (Sekhon & Saluja, 2011). As with all biologics, there is a need for long-term monitoring of patients receiving biosimilars to detect differences in safety or efficacy profiles that may emerge over time, as more patient experience is gained. Advanced practitioners should be aware of applicable risk-management plans and of the nature and timing of post-marketing studies required for these biosimilars, and they should consider requesting that their organizations support these efforts by providing relevant updates on the biologics used in their practice.

Advanced practitioners play a key role in educating nurses as well as patients. Nurse education in regard to biosimilars should include elements designed to help nurses understand key differences between generic drugs and biosimilars and be aware of any differences between the biosimilar and its reference product that will impact clinical use of the product, such as differences in the variety of approved indications, delivery devices or container closure systems, delivery/routes of administration, and storage and handling (FDA, 2012c). Understanding the differences in substitution practices between interchangeable and noninterchangeable biosimilars will also be important (Sekhon & Saluja, 2011).

Finally, nurses play a key role in collecting and capturing patient-reported AEs. Accordingly, nurse education should emphasize the importance of precise documentation of the administered product and any substitutions to support accurate attribution of AEs to the correct product. Patient education should emphasize self-monitoring for AEs, as well as any differences between the biosimilar and reference product in administration, handling, and storage.

## CONCLUSIONS

Biosimilars, a new class of biologic therapeutics, may enter the US health-care market soon, potentially increasing access to important biologics for patients with cancer by lowering costs. These potential benefits should be considered in the context of the nature and production of biologics. Unlike generic small-molecule drugs, biosimilars are not identical to their reference product because of their inherent complexity and differences in proprietary manufacturing processes. The clinical effects, if any, of subtle structural differences between a biosimilar and its reference product may not be known when a biosimilar is approved or after manufacturing processes change, requiring ongoing safety monitoring as well with all biologics. The US policies on biosimilars continue to evolve, including policies on regulatory approval, interchangeability, and postapproval and long-term safety monitoring.

As the US oncology community prepares for the coming introduction of biosimilars, it is important for advanced practitioners to receive comprehensive continuing education on biosimilars to ensure public safety and traceability of these emerging agents. Advanced practitioners are in a position to educate other oncology nurse professionals to ensure safe practice related to the use of biosimilars (particularly regarding any differences in delivery methods), the potential for immune reactions, and the importance of documenting product substitutions. They also will play an important role in prescribing biosimilars and managing patients receiving these new biologics, as well as educating patients on the safe use of biosimilars and self-monitoring for AEs.

**Acknowledgment**

The authors thank James Balwit, MS, whose work was funded by Amgen Inc., for assistance in the preparation of this manuscript.
